# Supplementary material for: Self-Control, Openness, Personal Need for Structure and Compensatory Control Change: A Serial Mediation Investigation
Source: Behav Sci (Basel). 2024 Apr 23;14(5):352. doi: 10.3390/bs14050352 (PMC11118860; doi:10.3390/bs14050352)
Supplement: Supplementary file 1 [file behavsci-14-00352-s001.zip › behavsci-2938706-supplementary.pdf]

Personal Need for Structure Scale

Read each of the following statements and decide how much you agree with each according to your attitudes, beliefs, and experiences. It is important for you to realize that there are no "right" or "wrong" answers to these questions. People are different, and we are interested in how you feel. Please respond according to the following 6-point scale:

1 = strongly disagree, 2 = moderately disagree, 3 = slightly disagree, 4 = slightly agree, 5 = moderately agree, 6 = strongly agree

1. It upsets me to go into a situation without knowing what I can expect from it.
2. I'm not bothered by things that interrupt my daily routine.*
3. I enjoy having a clear and structured mode of life.
4. I like to have a place for everything and everything in its place.
5. I find that a well-ordered life with regular hours makes my life tedious.*
6. I don't like situations that are uncertain.
7. I hate to change my plans at the last minute.
8. I hate to be with people who are unpredictable.
9. I find that a consistent routine enables me to enjoy life more.
10. I enjoy the exhilaration of being in unpredictable situations.*
11. I become uncomfortable when the rules in a situation are not clear.

Note: * Item is reversed scored.

Self-control Scale

Using the scale provided, please indicate how much each of the following statements reflects how you typically are.

1 = strongly disagree, 2 = moderately disagree, 3 = not sure, 4 = moderately agree, 5 = strongly agree

1. I have a hard time breaking bad habits.*
2. I am lazy.*
3. I say inappropriate things.*
4. I do certain things that are bad for me, if they are fun.*
5. I refuse things that are bad for me.
6. I wish I had more self-discipline.*
7. People would say that I have iron self- discipline.
8. Pleasure and fun sometimes keep me from getting work done.*
9. I have trouble concentrating.*
10. I am able to work effectively toward long-term goals.
11. Sometimes I can’t stop myself from doing something, even if I know it is wrong.*
12. I often act without thinking through all the alternatives.*

Note: * Item is reversed scored.

Openness Scale (subscale from Chinese Big Five Personality Inventory)

The following statements describe people's personality traits. Please decide how much you agree with each sentence according to how well it matches your personality.

1 = strongly disagree, 2 = moderately disagree, 3 = slightly disagree, 4 = slightly agree, 5 = moderately agree, 6 = strongly agree

1. My mind is often filled with vivid images.
2. I am a person who takes risks and is brave enough to break the rules.
3. I like to go on adventures.
4. I have a strong curiosity about many things.
5. I am more adventurous than others.
6. I am eager to learn new things, even if they are not related to my daily life.
7. My imagination is quite fertile.
8. I am willing and able to accept new things, new perspectives and new ideas.

Compensatory Control Change Scale

Questions of the compensatory control change scale are presented as follows (All of option B represent compensatory control and vice versa of options A):

1. Do you have a habit of hoarding before/after the outbreak? (A. no; B. yes)

2. Your primary reason for shopping before/after the outbreak: (A. I want; B. I Need)

3. Your trip packing habits before/after the outbreak: (A. I prefer to go light and pack as little as possible; B. I prefer to be well-prepared and bring as much as possible)

4. Do you set long-term goals before/after the outbreak? (A. I am more future-oriented, setting long-term objectives and working hard to achieve them; B. I am more present-oriented and attempt to do the right thing now)

5. Before/after the outbreak, your ideal occupation was: (A. IT Industry/self-employment/other; B. Further education/civil service, state-owned enterprises or institutions)

In each question, option A is assigned a value of 1, while Option B is assigned a value of 2. The score of the compensatory control change is the difference between before and after the outbreak (the value after the outbreak minus the value before the outbreak), with a total score of five questions ranging from −5 to 5. The positive score indicates more compensatory control behaviors after the outbreak than before, whereas the negative value indicates vice versa.
